# Greencoin as an AI-Based Solution Shaping Climate Awareness

**DOI:** 10.3390/ijerph191811183

**Published:** 2022-09-06

**Authors:** Hanna Obracht-Prondzyńska, Ewa Duda, Helena Anacka, Jolanta Kowal

**Affiliations:** 1 Department of Spatial Management, University of Gdańsk, 80-309 Gdańsk, Poland; 2 Institute of Education, Maria Grzegorzewska University, 02-353 Warsaw, Poland; 3 Department of Economics, Faculty of Management and Economics, Gdańsk University of Technology, 80-233 Gdańsk, Poland; 4 Institute of Psychology, University of Wrocław, 50-137 Wrocław, Poland

**Keywords:** smart city and artificial intelligence, climate change education, AI-based net zero solutions

## Abstract

Our research aim was to define possible AI-based solutions to be embedded in the Greencoin project, designed as a supportive tool for smart cities to achieve climate neutrality. We used Kamrowska-Załuska’s approach for evaluating AI-based solutions’ potential in urban planning. We narrowed down the research to the educational and economic aspects of smart cities. Furthermore, we used a systematic literature review. We propose solutions supporting the implementation process of net zero policies benefiting from single actions of urban dwellers based on the Greencoin project developed by us. By following smart city sectors, the paper introduces AI-based solutions which can enrich Greencoin by addressing the following needs: (1) shaping pro-environmental behaviors, (2) introducing instruments to reinforce the urban management process, (3) supporting bottom-up initiatives allowing to shape urban resilience, (4) enhancing smart mobility, (5) shaping local economies supporting urban circularity, and (6) allowing better communication with residents. Our research fills the gap in the limited group of studies focused on shaping climate awareness, enhancing smart governance, and supporting social participation and inclusion. It proves that AI-based educational tools can be supportive when implementing adaptation policies toward climate neutrality based on our proposed AI-based model shaping climate awareness.

## 1. Introduction

AI is considered today as a tool allowing to achieve sustainable goals [1,2]. Therefore, urban planning these days has evolved towards advancing the use of digital and AI-based tools to respond to urgent urban challenges [3]. Nevertheless, despite the increasing knowledge on the potential of artificial intelligence in shaping urban processes, the number of such AI-based tools remains limited [4]. At the same time, the role of the smart cities concept plays a key role in managing contemporary urban processes. However, to ensure the progress of its implementation, we need to consider climate change mitigation, shaping a healthy urban environment, and anticipating low-carbon technologies [5].

In response to such a challenge, there is a potential solution, according to Giffinger [6], in the smart city concept, which comprises all of these aspects. It additionally involves integrated data collection, sensing, networking and the impact of new social media, mobility, and travel behavior [7].

Based on the aforementioned considerations, the following questions arise:

- Can AI-based tools enable education on the net zero city, allowing to shape climate change awareness toward building healthy urban environments?
- Can such a solution, when embedded in the smart city policy implementation process, ensure pro-environmental behaviors of urban dwellers?

Therefore, the main aim of this research was to define possible AI-based solutions to be embedded in the Greencoin cybernetic system, designed as a supportive tool for smart cities to achieve climate neutrality. The assumption was to introduce solutions presenting the implementation process of climate neutrality policies that can benefit from single actions of urban dwellers and the potential impact of such an approach on urban resilience. When using Greencoin and educating by showing the results of joint efforts, we can engage residents and shape pro-environmental behaviors. 

Depending on the functionalities, Greencoin can influence all sectors of the smart city concept defined by Giffinger et al. [6,8]. Therefore, the main aim of the research was further fulfilled with the following sub-aims:

- Recognize existing AI-based tools that can be considered supportive while shaping pro-environmental behaviors in the following fields: (1) smart people—smart education; (2) smart mobility; (3) smart living—social participation; (3) smart environment—urban adaptability and resilience; (4) smart economy—circularity, sustainable development, and green economy; (5) smart governance—digital twin concept and improved communication.
- Evaluate solutions which can be considered influential on climate change and climate neutrality education in smart cities.
- Perform in-depth analyses of AI-based solutions recognized as the most beneficent and most suitable for the Greencoin design to introduce in the conceptual model.

To ensure we deliver a concept for a tool supporting urban sustainability, a smart economy, and a smart digital city, we used Kitchenham’s [9,10] and the PRISMA [11] methodology and the SLR approach for our model creation.

Such a solution was designed with the aim to include AI-based functionalities to support the process of shaping the pro-environmental behaviors of urban dwellers. However, to become a tool enabling urban adaptability and the shift toward carbon neutrality, it needs to be embedded in the educational processes tackling all aspects of smart city development with the use of AI [6,8]. Therefore, we are designing a Greencoin tool that will operate based on an application so that, through its educational dimension, its action will bring social impact and behavioral change in terms of climate mitigation. Additionally, we will use the capacities and scope of technologies that allow to reach a larger target group of urban dwellers, anytime and anywhere, and support them in changing their behaviors towards ecological awareness and care.

**Figure 1:**
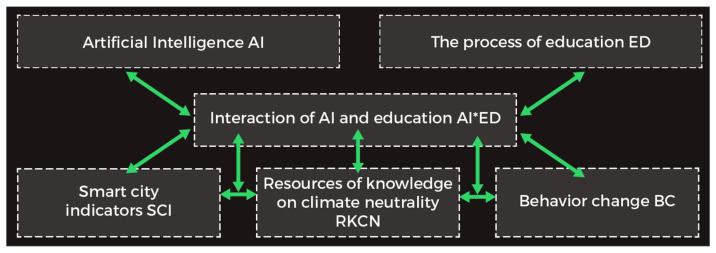
Research idea. Authors’ own elaboration.

Figure 1 illustrates the research idea. Based on the initial literature review, we assumed that artificial intelligence (AI) application combined with the education process (ED) could influence the shape of smart city indicators (SCIs), expand knowledge about climate neutrality (RKCN), and jointly influence pro-ecological behavioral change (BC). At the same time, the AI*ED interaction can affect the relationship between SCIs, RKCN, and BC. On the other hand, SCI, RKCN, and BC data can influence the AI*ED interaction, which is based on assumptions about AI self-learning. The results of our research can be included as Greencoin functionalities.

To respond to the questions and aims addressed above, the paper is structured as follows: Section 1 introduces the topic, Section 2 summarizes the theoretical framework and establishes the research gap, and Section 3 focuses on the research aims and methodology. Section 4 presents the empirical study results covering the case studies under the following topics: smart people, smart mobility, smart living, smart environment, smart economy, and smart governance. The crucial part is an introduction to the AI-based solution shaping the climate awareness model. Section 5 is devoted to the discussion, while Section 6 concludes the study.

## 2. Literature Review

Many authors have noted that an increase in the global mean temperature will lead to climate change—from heat and drought to more excellent spells and violent storms, more intense floods, forest fires, and plant extinctions—which requires a shift toward pro-environmental behaviors of urban dwellers [12]. Such changes cause adverse effects on urban life and human health, mainly through air pollution from the combustion of fossil fuels. There may be increased respiratory rate disturbances due to the increase in the frequency of smog (ground-level ozone) and airborne dust pollution. Ground-level ozone can damage lung tissue and is especially harmful to people with asthma and other chronic lung diseases [13]. There are also indirect effects related to climate-induced physical and social systems changes.

City dwellers exposed to adverse socio-behavioral and socioeconomic conditions become susceptible to environmental impacts and experience diminished health outcomes. There are relationships between the built environment (e.g., housing and transport infrastructure) and indoor air pollution and its effects on quality of life (physical and mental) [14]. Urban dwellers perceive the manifestations of climate change that can affect their physical and mental health [15]. Therefore, it is crucial to know and be aware of it and to change behavior to be more environmentally friendly, not only at the level of the local authorities but also at the individual level.

Urban areas today need to implement healthy city concepts, including low-carbon planning and design policies toward urban resilience. It can be achieved through improved public traffic systems, green open space planning, urban energy-saving initiatives, low-carbon economic strategies, ecological research, and the development of new technology applications that provide a model for climate neutrality and social engagement [16].

We argue that using all education methods with communication and information technologies, especially artificial intelligence, can reduce the environmental pollution in cities [4]. Therefore, there is a need for a solution which would allow for shaping climate change awareness. This can be achieved if such an AI-based tool is designed to serve the smart city concept, as it is proven that such an approach would need to move beyond urban cybernetics to tackle current challenges [3,4]. The idea of how to accelerate such an approach is described below.

### 2.1. The Role of the Smart City Concept

The smart city approach, as implemented worldwide, brings a creative mix of emerging technologies and open innovations that are changing the ways policy-makers develop strategies enhancing the quality of life of citizens [17]. It has introduced new opportunities for city administrators and stakeholders in various sectors to rethink the concepts of urbanization and the development of healthy cities [18]. Moreover, smart and healthy cities are associated with low-carbon economy solutions dependent on adequate resources, green building, and low-carbon technologies’ implementation, among others [19,20]. Information technology solutions can respond to the emerging challenges of the urban health environment [21]. Many demonstrators anticipating the paradigm shifts toward smart cities have been introduced worldwide [22,23]. However, the smart city approach must incorporate low-carbon technologies to move toward urban climate neutrality [5]. 

It proves that smart cities’ infrastructure should take into account the minimization of the specific problems of the modern world, namely unfavorable climate change and the need to diminish emissions [24]. For their better development and to counteract climate change, smart cities should use AI and the amenities of the Internet of Things (IoT). IoT means that some of the things we use can access the Internet (so-called intelligent/smart things). Anything connected to a processing unit (microcontroller) and connected to the Internet is considered an element of the IoT world. The first step in building an IoT device is figuring out how it will communicate with the rest of the world. 

### 2.2. Knowledge-Based Development Enhanced with AI

At the same time, the interest in using AI for urban innovation continues to grow [25]. The role of AI-based solutions and their potential in the process of shaping smart cities has been widely discussed [3,4,26]. It is no longer understood simply as collecting data and building from its comprehensive knowledge of the complex operation of a city. The infrastructure of modern cities needs to be supported by efficient information and communication technologies [27]. Sustainability-oriented urban projects consider digital tools as supporting smart development and management [28]. The current research on the smart cities approach emphasizes the need for unified assessment models for AI and big data processing within the urban space to mitigate climate change, e.g., by decarbonization, building energy efficiency, and estimating urban energy systems, but also, more importantly, to deliver tools for policy-makers to shape a high quality of life [29].

AI not only contributes to the development of smarter cities but primarily focuses on data analytics, education, environmental sustainability, health, security, transport, and urban management areas [30]. Different studies prove that there is a need to design environments by responding through technology to current educational challenges and enhancing learning processes [31,32,33,34,35]. Such an approach results from increasing the presence of AI in all aspects of human activity [35,36,37]; hence, researchers have provided comprehensive studies of technologies enhancing the inclusiveness and smart education of societies [36,37,38]. The increasing interest in artificial intelligence for innovative solutions and human-like intelligence results from its cognitive abilities, learning, adaptability, and decision-making capabilities [39]. They prove that AI technology can help education systems use data to improve educational equity and quality in the developing processes [35]. McArthur et al. [40] argue that the appropriate use of new technologies can lead to valued educational outcomes.

### 2.3. The Role of AI in Smart Urban Development

Artificial intelligence (AI) is the ability of machines, computer programs, and systems to carry out intellectual and creative functions, solve problems, draw conclusions, and make decisions. AI tools, including machine learning, deep learning, and predictive analytics, increase the ability to plan, learn, reason, think, and take actions, particularly aimed at finding ways to accelerate climate neutrality [41].

Studies have explored whether building artificially intelligent cities can safeguard humanity from natural disasters, pandemics, and other catastrophes [42], especially when climate change, overpopulation, and the squandering of resources pose problems of such magnitude that they require a change in the trend to mitigate their effects. Therefore, many solutions based on IoT can be found which allow the prediction of climate conditions [43]. Such possibilities can enhance society’s awareness and, on the bases of new digital solutions, educate people about the challenges of climate change as well as further establish a base for efficient urban development [44]. Rapid technological developments, including key features such as AI, the Internet of Things, 5G networks, and the increasing level of connectivity between everyday objects, contribute remarkably to reducing climate change [45].

With such potential, AI applications have also started to become an integral part of many urban services [30], especially when AI was recognized as a supportive tool for shaping city structures and the behavior of residents based on a rational decision-making system [46]. AI can establish a base for a people-centric approach where smart technologies are employed as tools to tackle social problems, address resident needs, and foster collaborative participation [47]. The social dimensions of evidence-based policy in a digital society have become the research focus of many studies [46]. However, the solutions should not be implemented as top-down initiatives imposed by the authorities and city administrators. Above all, they should be applied as bottom-up initiatives, involving the residents themselves, who best understand urban challenges [48].

### 2.4. AI-Based Tools Shaping Eco-Awareness

With this understanding, the intention of this study was to develop the concept of the Greencoin cybernetic system which, when enriched with AI-based technologies, will become an educational tool for urban dwellers, contributing to the process of shaping urban smartness. It aims to tackle the challenge of involving residents in the process of mitigating climate change and shaping urban adaptability. Therefore, our work is based on the research of Giffinger et al. [6,8], which defines six sectors of the smart city concept which we further link with the challenge of shaping climate-adaptive and net zero policies:

- Smart people—smart education: shaping pro-environmental behaviors with the use of AI-based tools.
- Smart mobility: shaping urban walkability and enhancing ecological transportation choices to diminish carbon emissions.
- Smart living—social participation: considering AI-based tools as supportive instruments in the process of urban management and using them while shaping a participatory approach and shaping climate neutrality policies.
- Smart environment—urban adaptability: developing AI-based tools responding to climate change while simultaneously supporting bottom-up initiatives allowing to shape urban resilience.
- Smart economy—circularity: searching for AI-based tools which can be considered supportive when shaping local economies to be based on sustainable growth, short supply chains, and local products and services as well as a circular economy.
- Smart governance: AI-based tools embedded in the digital twin concept, allowing better communication with residents and explaining urban processes.

### 2.5. Research Gap and Motivation

An initial Scopus search was conducted before the research was structured. Its results allowed us to identify a current research gap in the literature to be filled. Previous studies have proved that while initial research could be partially used in the empirical analysis, it is indeed a useful tool to identify research gaps for further, more detailed research investigations (e.g., [49,50]). Moreover, the Scopus database was chosen for the detailed research analysis because it is considered representative, reliable, and scientifically grounded and covers reputable publishing houses such as Emerald, Elsevier, IEEE, T&F, and Springer [51,52,53].

**Figure 2:**
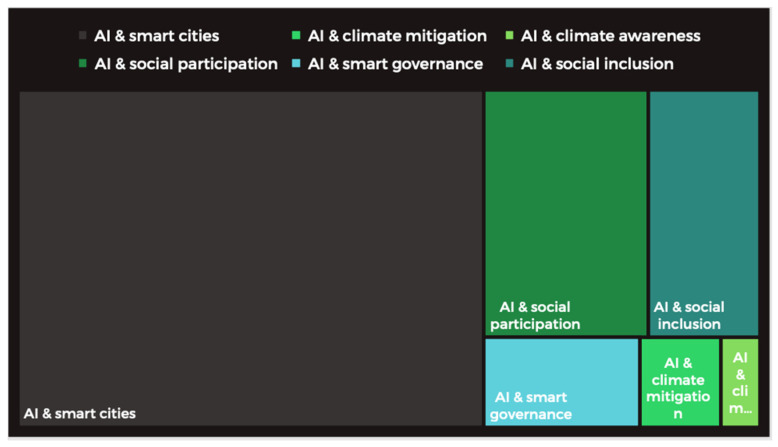
Research gap presenting the limited number of climate-related papers embedded in smart city concepts employing AI. Authors’ own elaboration based on Scopus keywords search.

This initial work has proven that AI-based tools are widely discussed in the context of the implementation of smart city policies. A random search of keywords related to the scope of research presented many uses of artificial intelligence defined so far; there is a limited group of studies introducing the possibilities of using AI-based tools for mitigating climate changes (33), shaping climate awareness (16), supporting social participation (185), shaping smart governance (64), and social inclusion (126). The proportions are presented in Figure 2. It shows a gap and the importance of developing AI-based tools in the urban context as their role in shaping sustainability has been proven [2].

## 3. Methods

**Figure 3:**
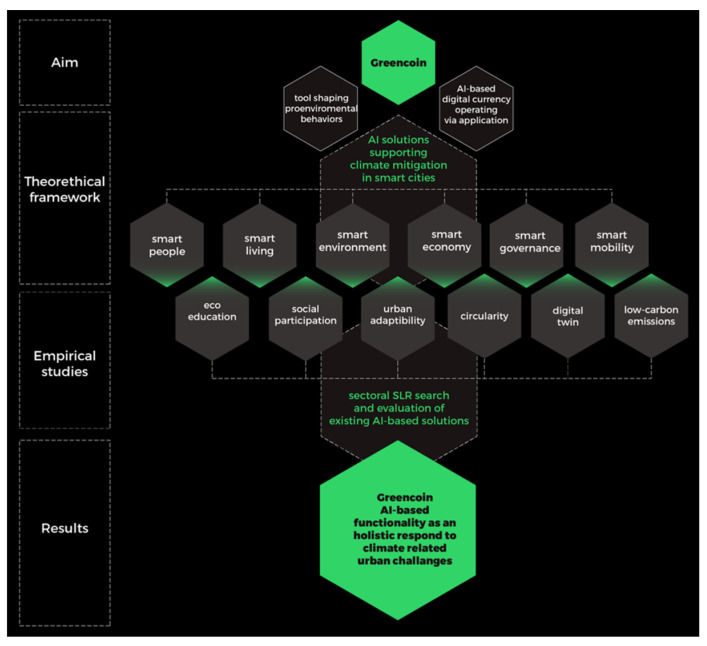
Research format. Authors’ own elaboration (based on methodological approach of Giffinger et al. [6]).

The paper describes the eight steps of research, while the study aims are linked with recognizing the useability of AI-based tools in all sectors of the smart city concept defined by Giffinger [6]. Figure 3 shows the general aim of the study, the methodological approach, and the research process itself, and it explains the structure of empirical studies.

The methodological approach is based on Kamrowska-Załuska’s study [54] evaluating the potential of AI-based solutions in urban planning. We narrowed down the study to the educational aspects of a smart city. We used qualitative methods [55], such as a systematic literature review [9,10,11], critical literature analysis, the method of competent judges [56,57], case studies [58], and the construction of a theoretical model based on SLR.

### 3.1. Model Building

**Figure 4:**
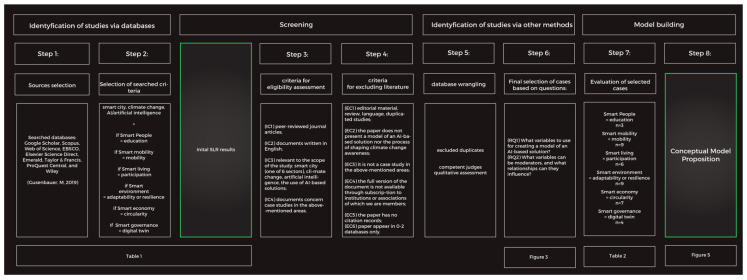
PRISMA application flowchart introducing the methodological approach (adapted from Yasin and Abbas [59] and Page et al. [11]).

Our study is qualitative by nature and is based on a review and analysis of the available secondary data. Our systematic literature review was based on the works of Kitchenham [9,10] and Page et al. [11], with the study flowchart depicted in Figure 4. We carried out the following steps to build a named theoretical model. Diagram 1 allows for navigating through the eight-step process. We built the model building procedure concerning checklist items (Appendix A).

### 3.2. SLR to Search for AI-Based Tools Embedded in the Fields Defining the Smart City Approach

The first step determined the data sources and search strategy. In the initial phase of the SLR, we used the following databases: Google Scholar, Scopus, Web of Science, EBSCO, Elsevier ScienceDirect, Emerald, Taylor & Francis, ProQuest Central, and Wiley [60].

### 3.3. Selection Criteria

The second stage of model construction defined the search criteria. Following Kitchenham [9,10] and Page et al. [11], we adopted keywords for the literature search through critical literature analysis and substantive discussion among the authors and defined six separate search queries corresponding to the sectors of the smart city concept [6,8] (see Table 1, rows 1–3).

In the third stage, we established criteria for including more literature items, as follows:

- (IC1) Peer-reviewed journal articles;
- (IC2) Documents written in English;
- (IC3) Relevant to the scope of the study: smart city (one of 6 sectors), climate change, artificial intelligence, the use of AI-based solutions;
- (IC4) Documents concerning case studies in the areas mentioned above.

In stage four, we defined criteria for excluding literature items:

- (EC1) Editorial material, review, language, duplicated studies;
- (EC2) The paper does not present a model of an AI-based solution nor the process of shaping climate change awareness;
- (EC3) It is not a case study in the areas mentioned above;
- (EC4) The full version of the document is not available through subscription to institutions or associations of which we are members;
- (EC5) The paper has no citation records;
- (EC6) The paper appears in 0–2 databases only.

In step five, to guard against the risk of bias—that is, a systematic error that can lead to erroneous results and conclusions in the design, conduct, or analysis of a study—we first excluded duplicates and then used the method of competent judges [56,57,61]. The case study selection procedure, inclusion and exclusion criteria application, and research questions’ formulation were carried out during semi-structured discussions between the authors of this paper and four experts in the field.

### 3.4. Initial SLR Results

Table 1 describes the initial results from the above-mentioned process. 

Row 1 represents smart city sectors, and row 2 consists of generic keywords relevant to the scope of the study: smart city, climate change, and artificial intelligence. As presented in rows 4–12, the Google Scholar database allowed us to find the highest number of case studies corresponding with the keywords, and the most limited number was found on the Web of Science. The blue fields show the concepts with the highest number of publications in each group. The table shows a limited number of studies on climate-related research using AI embedded in the smart city concept. The most common area of focus in the studies was on using AI-based solutions in shaping smart mobility, which is why we have identified it as the best explored topic. However, the field of smart mobility is inextricably linked to the other areas analyzed, which is why we have included it in our analysis.

### 3.5. Case Studies Analyses

In stage six, we focused only on AI-based tools embedded in the smart city concept that can be considered supportive in the process of (1) mitigating climate change, (2) shaping climate neutrality awareness, and (3) enhancing pro-ecological behaviors. To build a model, we defined detailed (compared to those addressed in the introduction) research questions. In the first review stage, we clearly defined the detailed research questions (RQ1) and (RQ2) as the points to be answered:

- (RQ1) What variables can be used for creating a model of an AI-based solution?
- (RQ2) What variables can be moderators, and what relationships can they influence?

Stage seven was related to data extraction and analysis and selected items. The summary of the evaluation is in Table 2. Variables for the model, such as dependents, independents (predictors), and possible moderating variables, were selected based on theoretical analysis. Following Kamihira and Yasuoka [62], we presented the model descriptively in a graphical diagram with the variables extracted. We assume that our theoretical model will be empirically verified during an experiment among city residents using the AI application in the future.

From the 42 selected articles, we identified a total of 3 main dimensions as latent variables and 18 latent variables as sub-dimensions (see Table 2). Latent variables are not directly observable and are usually measured by various indicators, such as questionnaires composed of items that can be expressed by numbers or symbols [63]. We then subjected the extracted variables to retrospective analysis concerning the research questions. Based on the study and substantive discussion among the group of competent judges, we distinguished three categories of variables, which we used to construct a conceptual model:

(1) Based on specific values of independent variables (IMP; e.g., EDU, DEM), information about the average values of correlated dependent variables can be predicted (assumption RA1). Not every independent variable implies causality, i.e., an effect on the dependent variable; there may be so-called apparent correlations. One can establish causality through an experiment.
(2) A moderating variable (e.g., AIT = EDU*AIT) affects the strength of the relationship between the dependent and independent variables (assumption RA3). It can weaken or strengthen the effect between the independent and dependent variables if found to be significant [63].
(3) As the dependent variable, we chose the global impact on climate mitigation in a smart city (ICMSC). The dependent variable (ICMSC) is predicted, and its value depends on changes in the independent variable (IMP). They can be related to behavioral change, a step toward climate neutrality and well-being [64] (assumption RA2).

The most general form of the model we propose is as follows.

**Figure 5:**
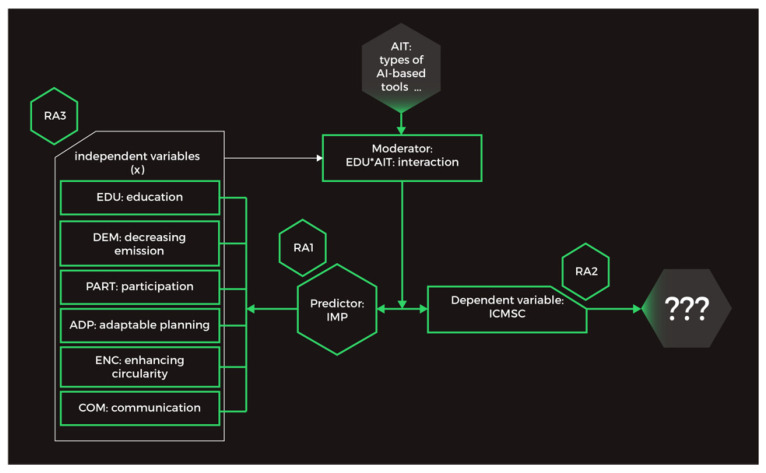
The initial assumptions for building the model. Authors’ own elaboration.

In stage eight, the initial assumptions of the theoretical model were embodied in the cybernetic project (Figure 5), which incorporates the functionalities of the Greencoin project.

## 4. Results

### 4.1. Case Studies Evaluation

Table 2 briefly introduces the main research findings from the SLR search grouped by case study analytical criteria. While the more extended evaluation of the cases selected for in-depth analysis is depicted below, the table itself depicts the following:

- Columns 1 and 2: sectors of smart cities and the keywords used in the SLR search;
- Column 3: most common aims of the research focusing on the role of AI and its relevance to the sectors of the smart city concept;
- Column 4: the analyzed cases;
- Column 5: summary of commonly used AI-based solutions in each smart city sector;
- Columns 6 and 7: the potential and limitations of such tools.

**Table 2:** The results of case studies’ analysis and evaluation. Authors’ own elaboration.

Independent Variables				Moderators	Dependent Variables		
Smart City Concept: Fields of Use		Keywords	Aims and Range	Research Studies	Types of AI-Based Tool (AIT)	Impact on Climate Mitigation in Smart Cities (ICMSC)	Limitations
1	Smart people	Education (EDU)	Human resources, teaching and learning, knowledge transfer	[30,65,66,67]	Interactive tools, AI and neural networks, machine learning (INTERAI)	Shaping pro-environmental attitudes and behaviors through a system of suggestions, encouragement, feedback, and positive reinforcement; solving challenging social environmental problems (SPAB)	Particular tested tools and solutions that can be easily implemented in the wider educational system
2	Smart mobility	Decreasing emission (DEM)	Traffic analyses, traffic capacity improvement, urban flows optimization, energy planning models, connectivity—spatial and social—autonomous mobility	[68,69,70,71,72,73,74,75,76]	IoT, satellite, cloud computing, AI/ML hybrid models (IOTAI)	Traffic and emission reduction; decarbonization; traffic optimization; mobility behavior change; emission awareness; eco-mobility promotion (TER)	More focused on optimization than behavioral change; often data visualization only; lack of social engagement mechanisms
3	Smart living	Participation (PART)	Indirect participation, understanding behavior patterns, social impact reflecting climate change visualization	[5,77,78,79,80,81,82]	Sensors, networks, artificial intelligence, applications of the Internet of Things, digital platform software (IoT platform), FI (Future Internet)-WARE Blockchain (SNAI)	Visualization of individual impact on climate change and understanding behaviors; influencing behavioral change on an urban scale (VISI)	Direct participation; coherent approach; integration of digital platforms relevant to sustainable goals
4	Smart environment	Adaptable planning (ADP)	Impact assessment, extreme weather conditions prediction, environmental and risk management, negative impact reduction	[73,78,83,84,85,86,87,88,89]	Sensors, knowledge-based intelligent systems, intelligent stochastic simulation models, genetic algorithms, evolutionary computing and spatial DNA (SKIS)	Urban microclimate improvement; efficient land use decarbonization; emission reductions prediction; urban energy, water resource, and waste management; environmental restoration (UMI)	Lack of integration; data collected sectorally; rarely provides base for strategic planning; limited educational impact—lack of integrated interactive data visualization platforms
5	Smart economy	Enhancing circularity (ENC)	Circular economy, sustainable development goals, transition to smart and green economy, eco-growth, low-carbon economy, green IoT, sustainable models of smart cities	[90,91,92,93,94,95,96,97]	Green IoT, UAVs (cameras, sensors), ICT 5G (such as 5G, beyond 5G, and sensors), radio frequency identification, new big data analysis based on AI-related tools, artificial neural networks, agent-based models, cloud-based services (GIOT)	UAV coordination in the cities with smart sensory data; IoT and UAV integration to decrease energy consumption, improve connectivity, and reduce pollution; agile adaptation to urban challenges (IOTU)	IoT intense energy consumption; personal data collection and analysis; data latency; fixed UAV trajectory; potential information invalidity; data gaps or bias
6	Smart governance	Communication (COM)	Smart governance (participation), participation in decision making, public and social services, transparent governance, political strategies and perspectives, ‘sharing economy’, AI for digital twin	[58,82,98,99]	App-based management, bots and artificial intelligence supporting social platforms activity, mobile AI application solutions (ABA)	Smart mobility approaches driven by big data strategies to address climate change; urban services mitigating climate change; modular platforms—collecting information from a wide range of sources to create more awareness about climate change resilience (SMA)	The global economic crisis and key messages to find a new role for urban planning in sustainable cities; limitations of case study research findings regarding generalization and application of findings: Budget limitations Need for more supporting infrastructure Lack of smart cities on short-term mindsets Lack of political will Lack of stakeholder support

#### 4.1.1. AI for Eco-Education

Introducing solutions that meet the needs of city dwellers while limiting climate change requires awareness and knowledge of how human activity affects the environment. Moreover, education is not only limited to making city dwellers more aware and strengthening their level of knowledge. Increasingly more significant is the need to accept progressive technological development and to actively involve particular social groups in the education process (even if it requires a notable amount of work, time, and resources), cooperation, and commitment. Therefore, educational activities are one of the key elements shaping smart cities, which also evolve into smart education [67].

The improvement to education must be made on several different levels. First, efforts are needed to adapt formal education to the needs of cities transforming. Modern technology’s increasing domination of our lives should support the educational process at different levels of education, shaping the desired digital skills of pupils, students, and adult learners. However, people cannot be limited only to learning the passive use of virtual digital devices. Creative and comprehensive solutions using algorithmics, machine learning, and artificial intelligence form the basis for the development of cities following the pro-environmental lifestyle. The changing job market following the changing of cities requires more and more specialists, professions that quickly adapt to different living and working conditions, replacing digital cities with smart cities, and with them, smart residents [67].

Second, smart solutions should facilitate the learning process, making it more efficient. The new ‘Smart Education’ approach, supported by solutions based on artificial intelligence, is increasingly becoming the subject of scientific discourse and projects attempting to implement such solutions [65]. The need to replace traditional methods of teaching and learning is increasingly pointed out. An example of implementing this approach in pedagogical practice is using interactive AI-based tools in education. Concerning younger children, they can support the process of learning programming (control of robot toys); as for older students or adult learners, they can support the process of self-education through a system of feedback and the selection of appropriate, subsequent exercises based on current achievements [66].

At the same time, new technology based on artificial intelligence and machine learning should be resilient to the emerging challenges facing cities. One of them is to provide adequate infrastructure for the effective education of residents, taking into account access to diverse social groups, web traffic, and current technological advancements of digital infrastructure. The effects of the difficulty of ensuring the right quality of education became glaringly apparent during the COVID-19 pandemic, during which the rapid need to shift to remote learning highlighted such problems as social inequality in access to education, the difficulty of providing adequate technical conditions, and the maladjustment of learners to the new conditions of the learning environment [30].

#### 4.1.2. AI for Mobility

Transportation plays an important role in healthy cities as one of the most important components of urban systems [68]. Research has proven that the smart city concept plays an essential role while mitigating transport emissions and meeting reduction goals [69]. Therefore, understanding and innovations of data-driven smart mobility in achieving decarbonization in urban development are crucial [71].

As smart mobility represents one of the six dimensions of a smart city, it involves both environmental and economic aspects and needs both advanced technologies and virtuous behaviors of people [70]. Transportation systems and their efficiency have a significant implication on not only resilience but also economic output, the social cost of cities, and the health of urban residents [71]. Unsustainable commuting models influence healthy conditions and expose residents to several risks [72]. Thus, many researchers worldwide are seeking solutions to improve the quality of urban commuting systems at the governance level. Kamrowska-Załuska found [54] that they are mostly focused on urban traffic analyses, the capacity of transport networks, commuting corridors, and energy planning models.

The analyzed papers prove that data-driven smart cities have a potential to enable emission reductions and climate change mitigation [73]. Therefore, data and data-based solutions should be used to tackle climate issues such as transportation efficiency and changes in travel behavior [71]. In particular, the improvement and integration of travel modes and efficient transport management can reduce greenhouse gas emissions in urban traffic [71]. Nowadays, smartphones, Wi-Fi, transit counters, traffic sensors, and toll payment systems, among others, provide detailed mobility and population data. Understanding human mobility allows to shape smarter, demand-driven transportation systems [74].

Intelligent data-driven mobility can be used for efficient transport activity by changing the behavior of travelers, as in the city of Hangzhou in China [71]. The function of AI is to reduce greenhouse gas emissions, mainly decarbonization, as a factor of Sustainable Development Goal 11.2, which is to ‘ensure access to safe, affordable, accessible and sustainable transport systems for all’, and Sustainable Development Goal 13.2, to ‘take urgent action to combat climate change and their effects’ and ‘integration of measures to combat climate change into national policies, strategies and planning’ by reducing greenhouse gas emissions every year. The mentioned goals require a public data strategy with a holistic view of complex urban challenges and global climate change [71].

Data-driven smart mobility initiatives applied in urban areas involve multiple stakeholders but with insufficient openness and limited access to data sharing [71]. Big data and AI-based technologies allow for shaping sustainable transportation models when using indirect participation methods [71]. Fortunately, AI enables newer methods of urban data acquisition using IoT, mobile phones, crowd sourcing, and social media [73]. The case studies analysis proves the importance of the ‘Fourth Industrial Revolution’ (4IR) technologies such as the Internet of Things (IoT), big data, artificial intelligence, smartphones, and cloud computing, which are influencing urban development as they allow air quality monitoring, following urban flows, and improving quality of life by overcoming communication barriers [73]. Even blockchain frameworks are used to shape people-oriented smart mobility systems [74].

The major finding from the case study analysis is that not only do data play a vital role when reconstructing urban transportation systems when searching for optimization mechanisms, but a high correlation between social attributes and the economic awareness of urban dwellers while shaping alternative mobility services can also be observed [75]. Smart mobility is therefore largely permeated by ICT, used in both backward and forward applications, to support the optimization of traffic flows and also to collect citizens’ opinions about livability in cities or the quality of local public transport services [70]. Additionally, AI-based solutions allow real-time mitigation governance [73], which can lead to more efficient public transportation models and to changes in the mobility behavior of urban dwellers. Surely, the new possibilities of connectivity; exceptional processing power; and the growing role of emerging technologies such as AI, IoT, robotics, and self-driving vehicles will advance intelligent mobility in the coming years [76].

#### 4.1.3. AI for Social Participation

The smart city approach promotes the integration of climate strategies and encourages the participation of citizens [77]. Social participation in the process of mitigating climate changes calls for a stakeholder-centric approach [82]. The research proves that the engagement of stakeholders can be achieved while designing digital practices to promote positive change [5]. However, to keep the attention of urban dwellers, there is a need to involve stakeholders in co-creating value and to introduce solutions illustrating the benefits of applying a stakeholder-centric perspective. AI-based solutions surely help to process behavioral data and consequently to present the impact, which further allows to shape attitudes towards climate change.

Artificial intelligence can be used to analyze citizens’ opinions [80] with the use of, e.g., social media data [78,81]. Studies of the spatial pattern of the behavior of individuals, visualization of social networks, recognition and simulation of individual mobility, and sentiment analysis allow to define engagement tools that in turn allow to encourage pro-environmental behaviors. Digital services support and influence consumers’ decision-making and promote sustainable behavior. Ågerfalk et al. [5] demonstrated that when developing knowledge about socially sustainable behaviors, urban dwellers will likely continue such behavior in the future.

As found by Kamrowska-Załuska [54], self-organizing mechanisms can help to achieve sustainability and allow the evaluation of dynamic attributes on the spatiotemporal scale, namely the preferences, emotions, and satisfaction of individuals. They further allow indirect participation with a new type of analysis based on specific behavioral patterns and as such can provide more reasonable and accurate explanations for the evolution of the mechanisms of complex systems.

Thus far, the usability of AI as a participatory tool has been recognized mainly in creating warning systems [78] or improving transportation efficiency while managing mobility demand and routing [79]. Many important aspects of climate mitigation are still underdeveloped. Certainly, visualizations of predictions have been recognized in the literature as tools helping to understand behaviors and influencing behavioral change on an urban scale.

#### 4.1.4. AI for Urban Adaptability and Resilience

Artificial-intelligence-based applications are becoming meaningful tools for disaster prediction and management [78]. When analyzing selected case studies, we found AI as supportive in the following aspects:

- In terms of improving the urban microclimate and responding to the challenge of urban heat islands [73];
- In analyses of major environmental threats, e.g., flooding [84,85], heat waves, and air quality [73,88];
- In the enhancement of efficient land use management [89] and emission reduction and decarbonization [88,100].

New technology can indeed improve the robustness and adaptability of the urban environment and build a secure and resilient urban system of emergency risk monitoring based on integration and communication [83,86]. Based on the analysis of case studies, we can observe that disaster risk management is one of the most significant issues where the role of AI-based tools is sought. However, since AI as a predictive tool can be helpful while shaping resilience, most of the research is focused mainly on flooding issues.

Supplementary to the tools supporting urban resilience, AI-based solutions were recognized as an enhancement of the circular economy by responding to the challenge of, e.g., water resources [84] and waste management [87]. Additionally, they can help shape assessment tools to build climate awareness as they can enhance the analyses of the resilience of urban structures [86].

Based on the cases evaluated, we can learn that most tools use sensors, knowledge-based intelligent systems, intelligent stochastic simulation models, genetic algorithms, evolutionary computing, and spatial DNA. The predictive power of artificial intelligence plays a significant role when using such tools in shaping urban resilience. This approach can help improve urban microclimates, use land efficiently, reduce emissions, conserve resources, and restore the environment.

However, the research shows that the pilot solutions—both developed as theoretical concepts and implemented—respond only to some aspects of a resilient city. At the same time, the conception requires a holistic approach. The remaining challenge is the lack of integration resulting from data collected sectorally. Therefore, such tools are rarely a base for strategic planning and hence have a limited educational impact. The problem of fragmentation and the lack of integrated interactive data visualization platforms urgently need to be addressed.

#### 4.1.5. AI for Smart Governance and Digital Twin

The digital twin concept has appeared in the scientific literature and practice. It is a combined approach to new forms of modeling and analysis based on big data and machine learning/artificial intelligence that combines the capabilities of a virtual model, data management, analytics, simulation, system control, visualization, and information sharing. Such an approach is considered a potential solution for positive energy districts (PED) optimization, for example, because it requires the integration of various systems and infrastructures to obtain optimal interactions between buildings, stakeholders, mobility, energy systems, and ICT systems [98].

The case studies prove that artificial intelligence depends on various underlying conditions, such as the political system, geography, and technology diffusion. Intelligent solutions cannot be copied; therefore, the value of each field should be assessed differently. Technologies can be used in cities to empower citizens by adapting these technologies to their needs rather than adapting their lives to technology demands. Moreover, the meaning of a smart city is multifaceted. The critical point is that cities need to respond to changes in the context in which they operate [99].

AI presents the challenge of urbanization and sustainable cities and provides a better understanding of the entire energy flow in a city with the actual characteristics of all buildings, total energy distribution, production, economy, and other aspects. One can see this process in the Shiraz city project, a sustainable new city, and in the AI techniques necessary to create a sustainable smart area, such as using the high potential of natural resources, the built environment and natural components, new methods and technologies based on smart cities, and intelligent infrastructure, thus improving the long-term social and ecological health of cities [58].

AI can facilitate engagement and transactions between consumers and suppliers on the smart city platform and expand the user base. AI can help develop a sense of community that functions in both online and offline environments. However, in a smart city, the development perspectives of the participants are more interested in getting involved. When the platform is open to interaction, one can access valuable information and contribute, thanks to AI. Accordingly, if the public sector has an open approach and promotes public consultation, citizens are more likely to become co-creators in this process [82]. Based on the predictive power of AI-based tools, it should have the potential to support the development of digital twins concepts as they require simulation tools. However, the research shows a limited number of such solutions.

#### 4.1.6. AI for Circular Economy

In the cases analyzed, agglomerations appear as significant contributors to regional and national economic growth [94,95]. While the sustainable economic development of cities has been set as the 11th Sustainable Development Goal (SDG), ICTs, IoT, and AI could assist in the process of achieving this goal [91]. According to the analyzed case studies, logistics, transportation, and energy, among others, connected through the IoT, contribute to the new ‘sharing economy of Collaborative Commons’ [92] that is sustainable and efficient, despite potential economic slowdowns. Moreover, cities in low-carbon transition can diversify their pro-environmental actions and increasingly rely on technological advancements in creating low carbon economic value [90,93]. The case studies evaluation from Asia and Africa shows that city productivity and size, quality of infrastructure, and public institutions have a higher impact on economic growth than urbanization expansion does [94]. Allam and Newman [95] pointed out that smart cities highly correlate with AI and big data usage and that their GDPs grow exponentially while fossil fuel use is declining.

As the analyzed examples show, AI and machine learning enable the modeling of urban development, growth, and consumption scenarios (water and energy consumption, transportation, and waste production); optimize energy and resources use and systems efficiency (e.g., self-organizing maps and agent-based models); and facilitate decision-making processes [94,96]. For example, polycentric spatial structure analysis, fuzzy logic, and analyses on cluster linkages allow to evaluate capital and resource flows cross-regionally, as well as to capture economic and social cluster connections [95,101]. The studied cases show that AI-based tools allow for the analysis of huge datasets, collected and updated instantly, that capture temporal data variations with high precision and at a lower cost, supporting smart urban development [91].

Furthermore, in the analyzed case studies related to the smart economy, an emphasis was put on sustainable development goals, eco-growth, circular economy, green Internet of Things, transition to a green economy, and sustainable models of smart cities [91,93]. Inter alia, big data analysis based on AI tools, green IoT (GIOT), UAVs, agent-based models, and cloud-based services are the instruments allowing to enhance the circular green economy [90,97]. Moreover, agile adaptation to various urban challenges is possible with the use of smart technologies—e.g., IoT and UAVs’ broad coordination and integration support a decrease in energy consumption and improve connectivity, coordination, and collaboration at a lower cost [96,97]. However, according to the literature, there are certain limitations associated with the use of AI in a green economy—for example, concerns related to personal data analysis and use (privacy concerns), data latency, potential information invalidity, and data biases and gaps as well as IoT intense energy consumption [91,95] are important issues to be addressed.

The performed case studies analysis shows that a sustainable green economy assumes resource availability and reusability for now and in the future, built as an interconnected, long-lasting ecosystem that derives advantages from AI, IoT, and data analysis and ensures access to smart public infrastructure, healthcare, energy, and transportation [92,93,96]. For example, the green Internet of Things not only provides higher economic efficiency by covering various urban systems, including communication, environmental preservation, surveillance, pollution, and transportation, but also allows the transmission and analysis of collected data through reliable and environmentally friendly Industry 4.0 [97]. To conclude, based on the performed cases’ study, ‘sustainable’ often goes hand in hand with ecological in the literature in the context of a circular economy, where AI-based tools could play a vital role in developing smart cities, ensuring their competitive advantage and long-lasting sustainable growth.

### 4.2. The AI-Based Solution Shaping Climate Awareness Model

**Figure 6:**
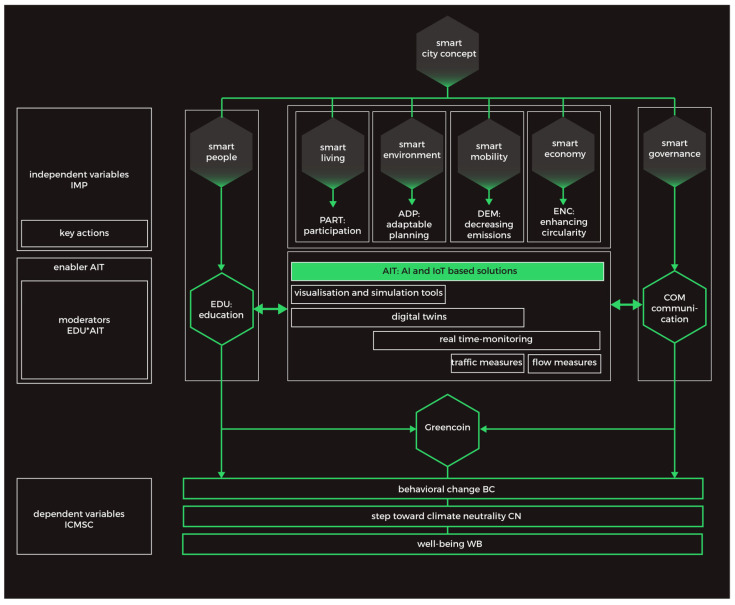
The AI-based solution shaping climate awareness model. Authors’ own elaboration.

Based on the SLR method and the analysis of the described AI applications, we propose the following model (Figure 6). The conceptual model for the AI-based solution responds holistically to all six sectors of the smart city concept. The six independent variables (smart city indicators), namely smart people, smart mobility, smart living (social participation), smart environment (urban adaptability), smart economy (circularity), and smart governance, affect BC, CN, and WB through ED and AI. Therefore, it can be considered a supportive tool influencing behavior change (BC), leading a move toward climate neutrality (CN) as a pre-dependent variable. Such an approach should enable a response to the need to shape healthy cities offering well-being (WB) of urban dwellers (dependent variable: ICMSC).

To achieve such results, the research proves that among the independent variables, two of them, namely education (EDU) and communication (COM), play a vital role in successfully implementing AI-based solutions. Smart people supported by smart governance are key when working toward climate neutrality. However, they should be supported by AI-based tools in participation (PART), adaptable planning (ADP), decreasing emissions (DEM), and enhancing circularity (ENC) as such moderators can enable change toward raising climate awareness and climate mitigation (EDU*AIT). Among all the studied solutions, we found AITs which play a crucial role in such a process: visualization and simulation tools, digital twins, and real-time monitoring tools including traffic and flow measures.

The subscales of the dependent variable, such as the global impact on climate mitigation in the smart city (ICMSC) related to behavioral change, a step toward climate neutrality and well-being, should cover the following key dimensions (Table 2):

- SPAB: shaping pro-environmental attitudes and behaviors through a system of suggestions, encouragement, feedback, and positive reinforcement and solving challenging social and environmental problems;
- TER: traffic and emission reduction, decarbonization, traffic optimization, mobility behavior change, emission awareness, and eco-mobility promotion;
- VISI: visualization of individual impact on climate change, understanding behaviors, and influencing behavioral change on an urban scale;
- UMI: urban microclimate improvement; efficient land use decarbonization; emission reductions; prediction; urban energy, water resource, and waste management; environmental restoration;
- IOTU: UAVs coordination in cities with smart sensory data, IoT and UAVs’ integration to decrease energy consumption, improvement of connectivity, reduction in pollution, and agile adaptation to urban challenges;
- SMA: smart mobility approaches driven by big data strategies to address climate change, urban services mitigating climate change, and modular platforms collecting information from various sources to create more awareness about climate change resilience.

Additionally, as moderators, there should be AI-based tools (AITs) as well as their interaction with the education (AIT*EDU) that cities need today (Table 2):

- INTERAI: interactive tools, AI and neural networks, and machine learning-based solutions;
- IOTAI: IoT, satellite, cloud computing, AI/ML, hybrid models of AI and neural networks, and machine learning;
- SNAI: sensors, networks, AI, IoT-based applications, digital platform software (IoT platform), FI (Future Internet)-WARE, and blockchains;
- SKIS: sensors, knowledge-based intelligent systems, intelligent stochastic simulation models, genetic algorithms, evolutionary computing, and spatial DNA;
- GIOT: green IoT, UAVs (cameras and sensors), 5G ICT (such as 5G, beyond 5G, and sensors), radio frequency identification, new big data analysis based on AI-related tools, artificial neural networks, agent-based models, and cloud-based services;
- ABA: app-based management, bots and AI-supported social platforms activity, and mobile AI application solutions.

We assumed the AI*ED interaction to be a mediator [102], affecting the six smart city indicators and the strength of the relationship between the group of independent variables, BC, CN, and finally WB. On the other hand, these variables also influence the interaction of AI*ED, which results from the assumptions of AI systems. Our assumptions are inferred from the literature and our previous studies. We assumed that most variables are latent and unobservable directly. They will be measured using future tools (qualitative and quantitative), though such a coherent and holistic approach of the Greencoin project can be considered a response for the cities working toward climate mitigation.

## 5. Discussion

An overview of AI applications in cities provides valuable information for optimizing living conditions in the urban environment following sustainable social, economic, and environmental development [23,54,98]. The number of case studies found proved the increasing interest in the use of AI for urban processes [25], but our SLR search [9,10,11] showed that despite the recognized need to develop digital solutions to achieve urban climate neutrality [5], there is still limited evidence on tools that moved outside the theoretical or pilot phase.

However, most of the evaluated cases have the potential to respond to the need to shape the urban health environment [21]. The lesson learnt is that sustainability-oriented urban projects consider digital tools as supporting smart development and management [28]. The current research on the smart cities approach emphasizes the need for unified assessment models for AI and big data processing within the urban space to mitigate climate change through, e.g., decarbonization, building energy efficiency, and estimating urban energy systems, but also, more importantly, to deliver tools for policy-makers to shape a high quality of life [29]. AI not only contributes to the development of smarter cities but above all concentrates on data analytics, education, environmental sustainability, health, security, transport, and urban management areas [30].

EDU: The analyzed cases prove that AI technology can help education systems use data to improve educational equity and quality in the developing processes [35]. The research confirms that AI can help shape pro-environmental behaviors [12], as the tools we studied include application-based solutions both to support the process of education for raising the environmental awareness of urban residents and guide their green behavior change to obtain climate neutrality. Such possibilities can enhance public awareness and, based on new digital solutions, educate about the challenges of climate change and further establish a base for efficient urban development [44]. However, education will not be the answer to all the required climate change mitigation activities. It should cover a broad spectrum of climate issues and serve all smart city dimensions [6], where AI can be considered a supplementary tool.

DEM: All solutions recognized within the research prove that AI can help to foster a reduction in CO2 emission levels [71]. Examples include app-based ride hailing, smart mobility represented by tight monitoring of vehicle purchase and license distribution, web-based car-sharing platforms, green travel modes, UAVs documenting accidents and emergencies, and urban traffic coordination using smart sensory data. All of them have the potential to diminish climate impacts and influence climate awareness. However, when implementing AI-based solutions, we should be aware of the risks such as hypermobility.

PART: The analyzed cases offer practical suggestions that would increase the effectiveness of stakeholder engagement at the platform level as well as ways to facilitate new online and offline processes. The analyses prove that such solutions, however, should not be implemented as top-down initiatives imposed by the authorities and city administrators [48].

ADP: The research shows that artificially intelligent cities can safeguard humanity from natural disasters, pandemics, and other catastrophes [42]. The literature indicates that AI and neural networks can be employed to predict severe events and protect against them [24].

COM: Through its prediction power and visual impact, AI has the potential to influence social engagement and strengthen participation by allowing co-governance. It is a condition for the introduction of a zero-emission development while diminishing the negative impact on the environment [103]. However, the research shows that the potential of AI-based tools within this smart city dimension remains not fully recognized.

ENC: AI can help identify areas demanding support and integrate individual actions to solve complex urban problems for the implementation of net zero policies. Artificial intelligence and neural networks are also applied in the process of learning how to optimally design building structures, with good results in shaping behaviors that save resources, such as reducing energy consumption while maintaining thermal comfort conditions [76] or climate neutrality [77]. The research cannot, however, prove that the potential of AI to shape urban circularity is already recognized.

Our aim was to identify the best possible practices using AI that will contribute to the issuance of a city’s climate strategy, taking into account the ambitions set out and the city’s adaptability. We identified many solutions which can be considered supportive while shaping pro-environmental behaviors. This evaluation shows that AI-based smart technologies offer great potential for creating trusted resources and knowledge transfer. This knowledge, in turn, is a source for increasing residents’ awareness and making the right decisions when solving challenging social problems. We can therefore confirm that AI tools, including machine learning, deep learning, and predictive analytics, increase the ability to plan, learn, reason, think, and take actions, especially when finding ways to accelerate climate neutrality [41]. However, through knowledge transfer and strengthening of social–human capital, it is easier to make modifications for sustainable development and implement collaborative research programs and new big data analytics (e.g., geo-referenced data) based on AI-related tools.

## 6. Conclusions

Based on this case study evaluation, we can conclude that AI tools can impact urban dwellers’ attitudes and behaviors. Moreover, these technological tools, when appropriately used, can educate people and motivate them to contribute to building smarter cities. Regarding our research questions, AI-based tools can be considered enablers for urban adaptive planning through the educational functionalities of solutions embedded in the smart city concept, such as Greencoin.

AITs can further help to shape climate neutrality awareness and promote a low-carbon economy. However, to ensure this process, there is a need for a more strategic approach to introducing AI-based educational tools and defining linkages between these solutions and smart city policy implementation processes. Solutions such as Greencoin cannot operate successfully when not embedded in local policies. Their functionalities must be designed carefully with a clearly defined scope, specific objectives with priorities, a diversified portfolio of social actions, and within time frames.

Such findings make it possible to define Greencoin AI-based functionalities that are designed to serve all dimensions of a smart city and can shape pro-environmental habits. The proposed model involves a coherent approach bringing and defining linkages between the six smart city sectors proposed by Giffinger et al. [6,8], namely smart people (smart education), smart mobility, smart living, smart environment (urban adaptability), smart economy (circularity), and smart governance, and addressing them through/with its functionalities. The research analysis revealed that while AI-related tools, green Internet of Things, and cloud-based services contribute to the promotion of a circular economy, sustainability, and eco-growth, the limitations, such as IoT intense energy consumption, data latency, and data biases and gaps, also need to be handled.

One limitation of this study is the lack of verification of the spatial distribution of the existing solutions. It would be worth evaluating how similar solutions operate depending on the local context and level of city smartness. We will address these issues in future research, enriching the achieved results while developing Greencoin functionality in further steps. Another research limitation is the lack of a testing stage of the concept, but in the next phase, the Greencoin project will establish a 9-month testing phase of the application based on the conceptual research outcome. Only complete, available articles were considered according to the inclusion criteria to assess the risk of bias. Findings and conclusions at each stage of the study (including the SLR) were considered by a group of competent judges (the authors and four experts in the field). The final decision at each stage was made by mutual consensus.

The added value of this research is in the holistic methodological analysis of the most essential elements of a smart city, as well as the proposals of the practical implications of the results on the Greencoin application prototype to be built on the basis of the conceptual model proposed in the paper. These study results could be valuable for both researchers and policy-makers as well as social organizations, activists, and local associations interested in testing and implementing AI-based educational tools aimed at shaping climate awareness among urban dwellers. This is because they show which tools to use when looking for engaging mechanisms allowing for stronger social participation when increasing climate neutrality. They also emphasize the need for a more holistic and coherent approach when implementing such solutions, as climate change mitigation calls for joint efforts and more systematic interventions. The tools found within the research prove that they can play a vital role when presenting impact and change and visualizing the challenges. Data, visualization, and monitoring tools can support educational processes and lead to behavioral change.

## Figures and Tables

**Table 1:** Result of SLR search. Authors’ own elaboration.

Smart City Concept Covering Fields	Smart People	Smart Mobility	Smart Living	Smart Environment	Smart Economy	Smart Governance
Generic keywords responding to theoretical framework	smart city, climate change, AI/artificial intelligence					
Keywords (initial dependent variables)	education	mobility	participation	adaptability or resilience	circularity	digital twin
Google Scholar	386	3020	2700	667	144	388
Scopus	4	1	1	2	2	0
Web of Science	7	0	0	0	1	0
EBSCO	10	5	765	1106	594	754
Elsevier	25	47	31	48	21	25
Emerald	5	12	5	11	6	10
Taylor & Francis	4	6	3	6	8	11
ProQuest Central	61	295	148	103	79	95
Wiley	5	11	4	4	3	6
Selected studies (for case studies evaluation)	4	9	7	9	9	4

Note: Positions highlighted in blue indicate the concepts with the highest number of devoted publications, which have the broadest coverage in the subject literature.

## Appendix A

**Table A1:** Abstracts/papers items checklist * needed for systematic literature review based on Kitchenham et al. [9,10] and PRISMA 2020 [11].

Section and Topic	Item #	Checklist Item
**Title**		
Title, keywords	1	Identifying the report as a systematic review: *Greencoin as an AI-Based Solution Shaping Climate Awareness* *Keywords: smart city and artificial intelligence; climate change education; AI-based net zero solutions*
**Background**		
Objectives	2	Providing an explicit statement of the main objective(s) or question(s) the review addresses: Aim: *Our research aim was to* *define possible AI-based solutions to be embedded in the Greencoin project, designed as a supportive tool for smart cities to achieve climate neutrality.* General research questions: *Q1. Can AI-based tools enable education on the net zero city, allowing to shape climate change awareness toward building healthy urban environments?* *Q2. Can such a solution, when embedded in the smart city policy implementation process, ensure pro-environmental behaviors of urban dwellers?* Detailed research questions resulting from SLR: *(RQ1) What variables can be used for creating a model of an AI-based solution?* *(RQ2) What variables can be moderators, and what relationships can they influence?*
**Methods**		
Eligibility criteria	3	Specifying the inclusion and exclusion criteria for the review: Inclusion criteria for including literature items: *(IC1) Peer-reviewed journal articles;* *(IC2) Documents written in English;* *(IC3) Relevant to the scope of the study: smart city (one of 6 sectors), climate change, artificial intelligence, the use of AI-based solutions;* *(IC4) Documents concerning case studies in the areas mentioned above.* b. Criteria for excluding literature items: *(EC1) Editorial material, review, language, duplicated studies;* (*EC2) The paper does not present a model of an AI-based solution nor the process of shaping climate change awareness;* *(EC3) It is not a case study in the areas mentioned above;* *(EC4) The full version of the document is not available through subscription to institutions or associations of which we are members;* *(EC5) The paper has no citation records;* *(EC6) The paper appears in 0–2 databases only.*
Information sources	4	Specifying the information sources (e.g., databases, registers) used to identify studies and the date when each was last searched: *We based our data sources and search strategy on the use of many data sources such as Google Scholar, Scopus, Web of Science, EBSCO, Elsevier, Emerald, Taylor & Francis, ProQuest Central, and Wiley.* *We conducted a search looking for word chains that were a combination of keywords and terms, indicated in* Table 1*: smart city, climate change, AI/artificial intelligence, education, mobility, participation, adaptability or resilience, circularity, digital twin.*
Risk of bias	5	Specifying the methods used to assess risk of bias in the included studies: *To assess the risk of bias:* *Only full, accessible articles were considered according to the inclusion criteria;* *Conclusions and findings at each stage of the study (including the SLR) were considered by a group of competent judges (the authors of the article and four experts in the field);* *The final decision in each step was made by competent judges’ mutual consensus.* *Since we did not conduct a meta-analysis, we have not reported the summary estimate and confidence/credible interval.*
Synthesis of results	6	Specifying the methods used to present and synthesize results: *We used qualitative methods* [55] *such as a systematic literature review* [9,10,11],* critical literature analysis, the method of competent judges* [56,57],* case studies* [58],* and the construction of a theoretical model based on the SLR. The final results are presented descriptively, as well as in the form of tables and graphs.*
**Results**		
Included studies	7	Giving the total number of included studies and participants and summarize relevant characteristics of studies: Table 1 *and* Table 2; Section 4*. Results.*
Synthesis of results	8	Presenting results for main outcomes, preferably indicating the number of included studies and participants for each: Table 2*;* Section 4*. Results.* *4.1. Case studies evaluation.*
**Discussion**		
Limitations of evidence	9	Providing a brief summary of the limitations of the evidence included in the review (e.g., study risk of bias, inconsistency and imprecision): Section 5*. Discussion.* *(…) One limitation of this study is the lack of verification of the spatial distribution of the existing solutions. It would be worth evaluating how similar solutions operate depending on the local context and level of city smartness. Such issues will be included in future research, which will enrich the achieved results while developing Greencoin functionality in further steps.*
Interpretation	10	Providing a general interpretation of the results and important implications: Section 5*. Discussion.* *(…) The added value of this research is in the holistic methodological analysis of the most essential elements of a smart city, as well as the proposals of the practical implications of the results in the Greencoin application prototype.* Section 6*. Conclusions.* *(…) The contribution and a value of the research is that it proves the useability of AI-based tools for policy-makers, as the evaluated solutions show that AI-based tools help to encourage urban dwellers by shaping climate awareness and visualizing the impact. Therefore, Greencoin as an AI-based tool can be considered supportive not only for urban dwellers but also for decision-makers or educators recognized as beneficiaries.*
**Other**		
Funding	11	Specifying the primary source of funding for the review: NCBR: National Centre for Research and Development, grant number NOR/IdeaLab/GC/0003/2020-00.
Registration	12	Providing the register name and registration number.

* Source: Authors’ own elaboration, adapted from Kitchenham et al. [9,10] and PRISMA 2020 [11].
